# Effect of modality compatibility on dual-task performance in a more naturalistic environment

**DOI:** 10.1007/s00426-026-02238-0

**Published:** 2026-01-27

**Authors:** Piesie Akwasi Gyimah Asuako, Marie Mueckstein, Robert Stojan, Melanie Mack, Sina Alexandra Schwarze, Christine Stelzel, Yana Fandakova, Denise Nadine Stephan, Claudia Voelcker-Rehage

**Affiliations:** 1 Institute of Sports and Exercise Sciences, University of Muenster, Muenster, Germany; 2 International Psychoanalytic University, Berlin, Germany; 3 Center for Lifespan Psychology, Max Planck Institute for Human Development, Berlin, Germany; 4 Center for the Interdisciplinary Study of Gerontology and Vulnerabilities, University of Geneva, Geneva, Switzerland; 5 Department of Developmental Cognitive Neuroscience, University of Trier, Trier, Germany; 6 Institute for Psychology, University of Aachen, Aachen, Germany; 7 Department of Neuromotor Behavior and Exercise, University of Muenster, Muenster, Germany

**Keywords:** Dual-task, Modality compatibility, Classical laboratory, Driving simulator, Young adults, Ecological validity

## Abstract

The modality-compatibility effect describes multitasking-specific performance differences between compatible stimulus-response mappings (e.g., visual-manual and auditory-vocal) and incompatible mappings (e.g., visual-vocal and auditory-manual). The modality compatibility effect has been demonstrated in various studies on dual-tasking with classical laboratory experimental designs. However, it remains unclear whether the effect also exists in everyday-life dual-task situations, such as driving while performing an additional task. Here, we investigated modality compatibility effects under single-task and dual-task conditions and compared a classical laboratory setting with a more naturalistic driving simulator setting, in which participants acted as front seat passengers and were not required to operate the vehicle. We hypothesized larger dual-task costs under modality incompatible mappings compared to modality compatible mappings across settings, and a higher magnitude of the modality compatibility effect on dual-task costs in the classical laboratory setting than in the driving simulator setting. Participants (*N* = 31, age: 22.86 years ± 2.04, 21 women, 10 men) responded manually or vocally to visual or auditory stimuli (modality compatible mappings or modality incompatible mappings) in both settings. Results revealed greater dual-task costs for modality incompatible mappings than modality compatible mappings for reaction time only. However, overall dual-task costs did not differ between the classical laboratory and the driving simulator settings. Most importantly, there was a significant interaction effect between modality mapping and setting such that the effect of the modality incompatible mapping was more pronounced in the classical laboratory than in the driving simulator. These findings highlight that the magnitude of interference from modality incompatible mappings is weaker in more naturalistic settings compared to the classical laboratory. This suggests that the modality-compatibility effect may exist in real-world contexts, but not in the same way as in the classical laboratory.

**Supplementary Information:**

The online version contains supplementary material available at 10.1007/s00426-026-02238-0.

## Introduction

### Effect of modality compatibility on dual-task performance in a more naturalistic environment

Performing two tasks at the same time (dual-tasking) is a common part of daily life. For example, a passenger in a car might listen to spoken directions from a navigation system and give verbal instructions to the driver, while simultaneously looking at a map on their phone and manually entering the destination. In this scenario, dual-tasking may result in slower or more error-prone navigation (e.g., missing the destination), an inability to maintain the conversation, or both. Performance decrements when two tasks are performed simultaneously compared to performing each task separately are typically referred to as dual-task costs (Kahneman, 1973; Pashler, 1994; Pashler et al., 2008). According to previous studies on dual-tasking, dual-task costs can be influenced by the modality mappings of the component tasks (Hazeltine et al., 2006; Mueckstein et al., 2022; Ruthruff et al., 2006; Stelzel & Schubert, 2011; Stelzel et al., 2006). Here, modality compatibility refers to the similarity between the modality of the sensory input of a task (e.g., auditory or visual) and the modality of the sensory consequences of the task response, also called action effect (e.g., hearing what is being said as auditory consequence of speech; sensory consequence of a manual response is often mainly visual) (Stephan & Koch, 2016). Specifically, auditory input paired with vocal output and visual input paired with manual output are considered relatively compatible stimulus and response modality mappings. In contrast, auditory input paired with manual output and visual input paired with vocal output are considered incompatible stimulus and response modality mappings (Hazeltine et al., 2006; Stelzel & Schubert, 2011). It has been shown that dual-task performance, examined under classical laboratory conditions, involving auditory-manual and visual-vocal tasks (modality incompatible mappings), results in higher dual-task costs compared to conditions involving auditory-vocal and visual-manual tasks (modality compatible mappings) (Hazeltine et al., 2006; Levy & Pashler, 2001; Ruthruff et al., 2006; Schaeffner et al., 2018). Extending this observation to the driving scenario above, it is plausible to assume that performance costs are increased for incompatible mappings compared to compatible mappings, such as when the passenger types a message in response to the spoken directions from the navigation system (i.e. auditory-manual) or when looking at the map and at the same time verbally instructing the driver to their destination (i.e. visual-vocal).

Studies investigating dual-task performance attribute dual-task costs to processing limitations such as the information processing bottleneck (Pashler, 1994; Pashler et al., 2008) or the capacity-sharing model (Navon & Miller, 2002). Notwithstanding these theories, empirical data suggest that crosstalk can result in a severe deterioration of dual-task performance (Lien & Proctor, 2002; Miller, 2006). The concept of cross-talk proposes that overlapping central features in two tasks critically influence dual-tasking (Navon & Miller, 1987).

**Fig. 1:**
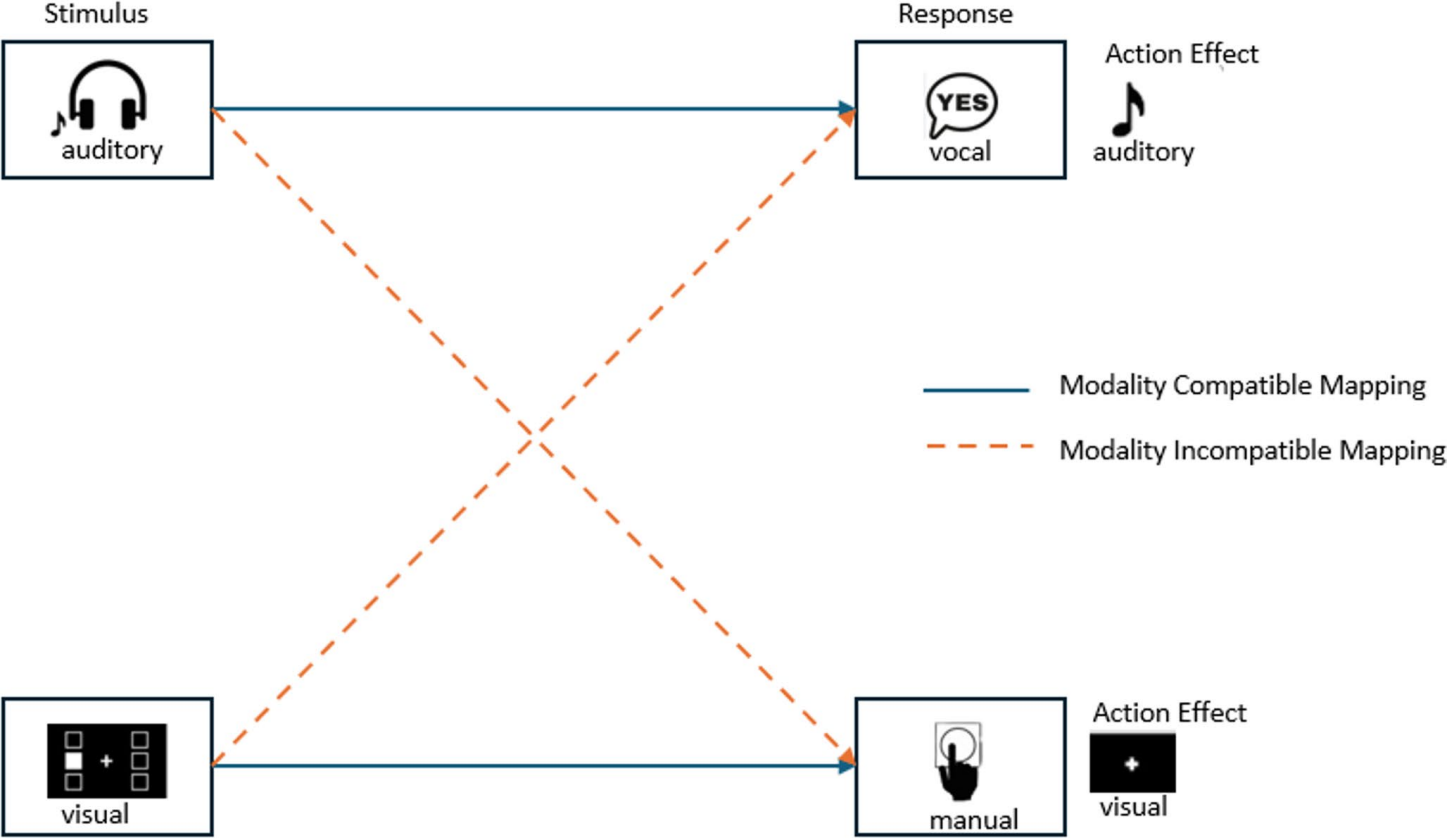
Modality compatibility effect in the context of dual-tasking. Note. The diagram illustrates the concept of modality compatibility in dual-task settings. Blue arrows represent modality compatible mappings, where the sensory modality of the stimulus aligns with the response and its expected action effect (e.g. auditory stimulus → vocal response → auditory effect; visual stimulus → manual response → visual effect). Dashed-orange arrows represent modality incompatible mappings, where the stimulus and response modalities do not align (e.g. auditory stimulus → manual response; visual stimulus → vocal response)

Modality-based crosstalk describes interactions between stimulus perception and the anticipated sensory effect of an action (cf. Figure 1). It occurs when the expected sensory consequence in one task relies on the same sensory modality as the sensory input in a concurrent task (Navon & Miller, 1987; Schacherer & Hazeltine, 2021). For example, in a modality incompatible dual-task situation, such as an auditory-manual task paired with a visual-vocal task, overlap in sensory input and sensory feedback across tasks (i.e., auditory) can increase interference and cognitive demands(Logan & Gordon, 2001; Schacherer & Hazeltine, 2021). Schacherer & Hazeltine (2021) demonstrated that the modality of experimentally induced action effects can modulate dual-task costs. Their findings showed that crosstalk significantly contributes to higher dual-task costs in modality incompatible conditions, as overlapping sensory feedback increases interference. In contrast, modality compatible mappings allow sensory input and response-related feedback to align within each task, reducing overlap between tasks and facilitating dual-task performance, for example, driving while conversing with a passenger.

It is important to note that much of the theories and research on modality compatibility have involved classical laboratory tasks. While these experiments provide precision and control, they have been criticized for their limited applicability to real-world settings (Chaytor & Schmitter-Edgecombe, 2003). Real-world settings are more complex at the sensory level, providing contextual cues that allow for multisensory integration, that is, the brain combines information from multiple sensory modalities (e.g. vision, hearing and touch) to enhance perception and task performance (Bailey et al., 2018; Cornelio et al., 2021; Jeong et al., 2024). This integration may mitigate dual-task costs in modality incompatible mappings by creating a unified sensory representation, thereby reducing crosstalk (Laurienti et al., 2006). Additionally, prediction of sensory outcomes, a fundamental aspect of intentional real-world behavior (Greenwald, 1970; Stephan & Koch, 2010), may further enhance performance in modality incompatible mappings. By predicting sensory outcomes, individuals can allocate attention and coordinate responses more effectively based on expected sensory consequences (Hommel, 2009; Prinz, 1997). For instance, a pianist can anticipate the auditory effects of pressing keys, allowing them to optimize their finger movements, despite the modality incompatible stimulus-response mapping. Therefore, while the pattern of the modality compatibility effect – defined as the performance difference between modality compatible mappings and modality incompatible mappings – may resemble that observed in classical laboratory settings, the magnitude of the effect may differ in real-world environments.

To address this gap, the study aimed to investigate whether modality compatibility effects on dual-task costs exist not only in classical laboratory settings but also in a more realistic everyday-life task, such as being a passenger in a driving simulator setting. Driving simulators have been widely used in dual-tasking research (Asuako et al., 2025; Bock et al., 2019b; Stojan et al., 2021; Strayer et al., 2015; Wechsler et al., 2018) as they allow researchers to study dual task performance in complex, realistic yet controlled settings. Furthermore, they provide realistic multisensory integration (Jeong et al., 2024) while ensuring control, safety, and ethical standards (Bock et al., 2019a; Calhoun & Pearlson, 2012). Moreover, simulators introduce real-world challenges such as sensory distractions and input variability, making them feasible for investigating dual-task performance in complex environments.

To test whether the modality-compatibility effect on dual-task costs observed in classical laboratory also manifests in real-world scenarios, we applied a 2 × 2 within-subject design. Participants completed modality compatible and modality incompatible single- and dual-tasks in two different experimental settings: a classical laboratory and a driving simulator. In the driving simulator setting, a cover story was introduced to enhance psychological fidelity, thereby increasing the realism and plausibility of the scenario (De Winter et al., 2007). Previous research has shown that the framing of a cover story can significantly influence participants’ interpretation and engagement(Hullman & Diakopoulos, 2011). Therefore, we aimed to compare this to a standard laboratory setting without the inclusion of a cover story in a counterbalanced order. Based on previous studies on modality compatibility (Fintor et al., 2018; Hazeltine et al., 2006; Mueckstein et al., 2022; Ruthruff et al., 2006; Schacherer & Hazeltine, 2020; Stelzel & Schubert, 2011; Stephan & Koch, 2016), we hypothesized that dual-task costs would be more pronounced under modality incompatible mappings compared to modality compatible mappings due to increased sensory interference in modality incompatible mappings (*main effect of mapping*). Additionally, we expected lower dual-task cost in the driving simulator compared to the classical laboratory *(main effect of setting)* due to the availability of multiple sensory modalities (e.g., visual input from seeing a car, auditory input from driving-related sounds) and contextual cues provided in the driving simulator (Roider et al., 2017). This multimodal input may rather facilitate dual-task performance by enhancing situational perception awareness (Deroy et al., 2016). Furthermore, we hypothesized that in the driving simulator, the integration of these sensory and contextual cues, combined with the increased task complexity, cover story and environmental dynamics, would enable participants to anticipate action effects more efficiently, even for modality incompatible mapping. This would result in reduced effects of modality incompatible mappings in the driving simulator than in the classical laboratory setting. Therefore, we expected the magnitude of the modality compatibility effect on dual-task costs to be more pronounced in the classical laboratory setting than in the driving simulator *(interaction effect of mapping and setting)*. With this research, we aimed to contribute to modality compatibility research by extending the investigation of its effect to a more realistic, everyday dual-tasking scenario.

## Methods

### Participants

To estimate the required sample size for testing the main hypotheses of this study, an a priori power analysis was performed with G*Power 3.1.9 software (Faul et al., 2007). The analysis was based on the effect size reported by (Mueckstein et al., 2022), who conducted a similar study in a laboratory-like environment (online-study) and reported an effect size of ƞ^2^ = 0.575. Using the following parameters for a within-subject ANOVA: α = 0.05, ß = 0.80, f = 0.25, number of groups = 1, number of measurements = 4, r(condition) = 0.5, the analysis indicated a sample size of 23 was sufficient to detect a moderate main effect and Setting * Mapping interaction. To account for potential attrition, we initially recruited 39 younger adults (mean age = 23.39 years ± 2.02, women = 27, men = 12) for the experiment. In addition, 13 participants (mean age = 22.31 years ± 1.93, women = 8, men = 5) were recruited post-hoc to counterbalance our original study design (cf. Figure 3). Recruitment took place via posters displayed in public spaces, the university’s weekly newsletter and students’ mailing list. Inclusion criteria were the following: aged 20–30 years, regular drivers (on average 1 time a week within the last 6 months), valid driving license, no mental illness, no uncorrected hearing and vision impairments, no orthopedic limitations, no past operations in the head area or brain, and not left-handed. Participants were compensated with three course credit points. Prior to their participation in the study, all participants were required to electronically sign an informed consent form via SoSci (www.soscisurvey.de). Due to technical issues with the recording of verbal data, 14 participants had to be excluded from the analysis. In addition, seven participants were excluded based on their error rates, for details, see section “Data Preprocessing”. Consequently, data from 31 participants (mean age: 22.86 years ± 2.04, women = 21, men = 10) were included in the final analysis. The study was approved by the ethics committee of the University of Muenster, Germany, and conducted in accordance with the ethical standards set forth in the Declaration of Helsinki (World-Medical-Association, 2013) and preregistered on AsPredicted (https://aspredicted.org/XDJ_RN2). At the time of pre-registration, experimental data from five participants had been collected, but not analyzed.

### Setting

The experiment was conducted using two different settings, including a classical laboratory setting and a virtual driving simulator setting.

#### Classical laboratory

**Fig. 2:**
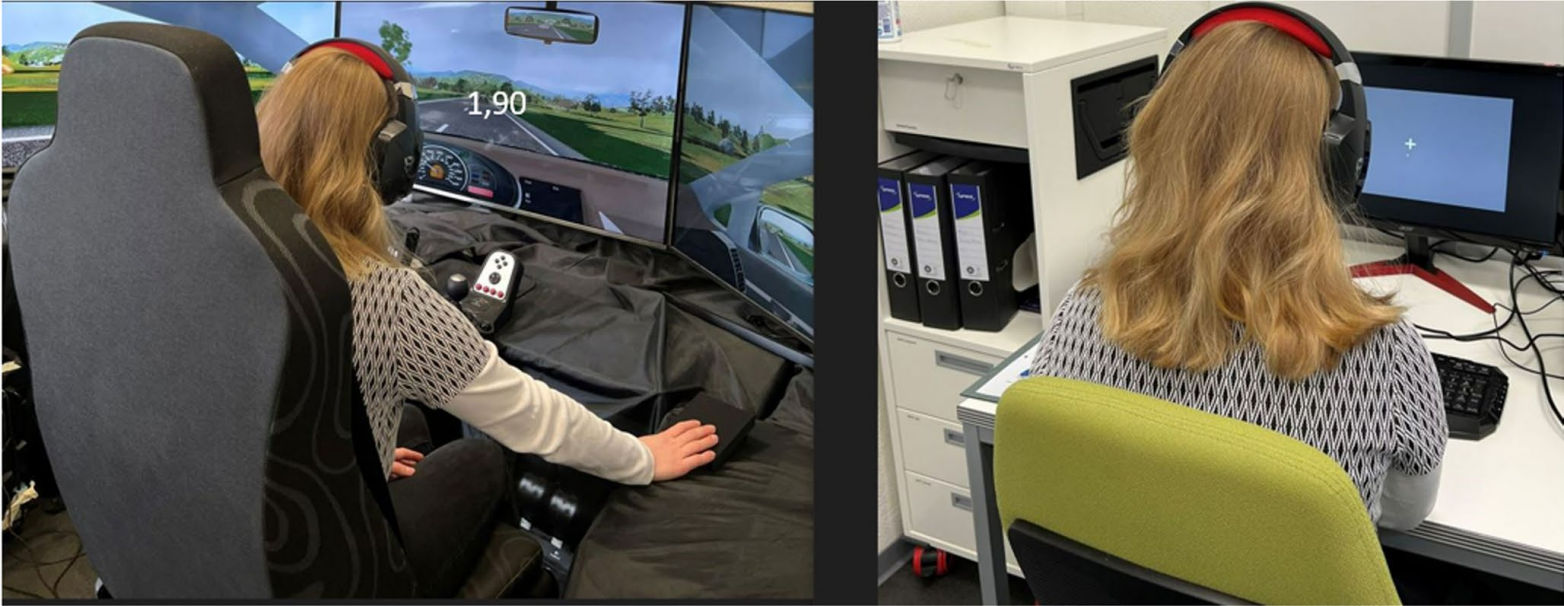
Driving simulator and classical laboratory settings. Note. The left image shows the driving simulator setting with a visual stimulus displayed in the center of the middle monitor. The right image shows the classical laboratory setting. An auditory stimulus is presented via the headset with a white fixation cross displayed continuously on the stationary screen

The classical laboratory setting was set up using the JsPsych library (version 6.1.0), an open source javascript-based tool. The experiment was hosted on the jatos online platform (version 3.5.4). A special built-in plug was used for the presentation and recording of both auditory and visual tasks (Mueckstein et al., 2022). Participants wore a headset for auditory task presentation, while visual tasks were displayed at the center of a stationary screen, extending from a Dell Latitude 5420 laptop (cf. Figure 2). Each participant completed the experiment using the Chrome browser.

#### Virtual driving simulator

The driving simulator setup comprised a VW Golf vehicle seat positioned in front of three 48” monitors, mounted at eye level, providing a 195° horizontal field of view. A modified Logitech G27 steering wheel (Logitech International S.A., Lausanne Switzerland) was mounted slightly to the left of the center monitor. The graphics, rendered by Carnetsoft version 8.0 (Groningen, The Netherlands), depicted a typical rural landscape with a winding road free of intersections and traffic lights. The scenery included a blue sky with clouds, mountains, animal enclosures, grassland, trees, traffic signs, petrol stations, construction sites and vehicles travelling at a constant speed in the oncoming lane (Wechsler et al., 2018). See Fig. 2 below for an impression of the setup.

The vehicle operated with automatic transmission and maintained a speed of 70 km/h (~ 19.4 m/s), eliminating the need for participants to steer, accelerate or brake. The participants’ car was positioned at a reasonable distance between two other cars, one ahead and one behind to mimic realistic driving conditions. Participants wore a standard headset with a microphone to receive auditory stimuli, which were superimposed with driving noise, and to record verbal responses. Verbal data was continuously recorded at 48,000 Hz using Audacity (version 3.3). Visual stimuli were presented centrally on the central monitor, and manual responses were made using a numeric keypad placed to the right of the steering wheel and adjusted to each participant’s reach. All keys except the numbers 5 and 6 were covered with black tape.

To enhance the ecological validity of the scenario, a cover story was introduced to the participant. They were asked to imagine themselves as passengers making decisions whether to ask the driver to refuel based on gas prices or to assess the length of a traffic jam to decide whether to stay on the route or take an alternative.

#### Stimuli

The stimuli and timing parameters were identical in both experiments, but their context varied by setting. In each trial, participants were randomly presented with either an auditory or visual stimulus for 600ms, followed by an interstimulus interval sampled between 5 and 6 s. The auditory task contained integers from 1 to 9 (excluding 5, which served as a reference value) and in the visual task decimal numbers from 1,10 to 1,90 (excluding 1,50, used as a reference) were presented (font color: white; size: 30 pt). Participants determined whether each stimulus was above or below the reference value. In the driving simulator setting, a contextual framing (cover story) was introduced in which auditory stimuli were presented as traffic jam lengths and visual stimuli as fuel prices, thereby enhancing the ecological validity of the setting. The numeric keypad was positioned to the participant’s right-hand side, requiring them to extend their right arm outward to respond (cf. Figure 2). In contrast, we did not include any contextual framing in the classical laboratory setting. Visual stimuli were displayed on a black screen, and a central white fixation cross remained visible during the presentation of auditory stimuli. Manual responses were made using a conventional keyboard positioned directly in front of the participant, similar to a typical desk setup (cf. Figure 2). Importantly, stimulus timing, task instructions, and overall response modality (vocal vs. manual) were held constant across both settings to ensure experimental comparability. However, the physical layout of the response devices for manual responses differed to reflect the respective environmental context.

#### Tasks

The experimental setup in both settings comprised three task types: single task, dual-task and task switching (not reported here). There were two conditions: modality compatible mappings and modality incompatible mappings. Within each condition, participants performed different task types, with instructions provided at the beginning of each block. In both settings, participants were instructed to respond as quickly and accurately as possible to each presented stimulus. They responded vocally by saying the German word “Ja” (English “yes”) whenever the presented stimulus was smaller than the reference value or the German word “Nein” (English “no”) whenever the presented stimulus was larger than the reference value and manually by pressing the key “J” with the index finger (“5” in the driving simulator) whenever the presented stimulus was smaller than the reference value or by pressing “K” with the middle finger (“6” in the driving simulator) whenever the presented stimulus was larger than the reference value.

#### Single-Task

Participants completed two blocks of each single task (auditory-vocal (AV), auditory-manual (AM), visual-manual (VM), visual-vocal (VV)). Each block consisted of 16 trials. The next block began immediately after the preceding block, following the presentation of an instruction.

##### Modality compatible condition 

Participants responded vocally to the auditorily presented integer (AV) and manually to the visually presented decimal (VM).

##### Modality incompatible condition

Participants responded manually to the auditorily presented integer (AM) and vocally to the visually presented decimal (VV).

#### Dual-task condition

Two dual-tasks were created by combining AV and VM (modality compatible condition) and AM and VV (modality incompatible condition). Within each dual-task condition, there was no overlap in either response modality or stimulus modality (i.e., participants were never presented with two visual stimuli or two auditory stimuli, nor did they respond using the same modality for both tasks). Each condition consisted of one block of 32 trials, with a 20-second break after the first 16 trials. Both stimuli were presented simultaneously (stimulus-onset asynchrony = 0), and participants were required to respond to both stimuli.

#### Motivation

Participants’ motivational state was assessed using the Dundee Stress State Questionnaire (DSSQ)(Matthews et al., 2013). The DSSQ is a validated self-report measure designed to evaluate task-related motivation, including engagement, distress, and worry. Although this measure was included to account for potential motivational influences on dual-task performance, we excluded it from the final model of the analyses to maintain parsimony as it did not change the pattern of the results (cf. Supplementary data; Appendix G for the results with motivation entered as a covariate).

### Procedure

The experiment consisted of two sessions with a rest day in between. All sessions were conducted at the Neuromotor Behavior and Exercise Laboratory at the University of Muenster, Germany. Before the first day of testing, participants received general information about the project and completed the informed consent, demographic, and driving questionnaires online via the SoSci platform.

In the initial version of the design, all participants performed the tasks first in the classical laboratory setting, followed by the driving simulator on the second day. In a later, counterbalanced version of the design, some participants started in the driving simulator on Day 1 and continued in the classical laboratory on Day 2.

In the classical laboratory setting, the order of the tasks was the same for all participants. They performed first the two single-tasks of one modality mapping (i.e., compatible or incompatible), followed by the corresponding dual-task. After that, they repeated the same procedure for the other modality mapping. The order of the modality mapping was counterbalanced between participants: 11 participants began with the incompatible mapping and 10 participants began with the compatible mapping. The post-hoc recruited sample was split into six participants who began with the compatible and four participants who began with the incompatible mapping (cf. Table 1). Before the start of each task block, the specific task instructions were presented auditorily and visually to the participants. The instructions were pre-recorded by the same voice as the auditory stimuli were recorded. Participants were not instructed to prioritize one task over another to enhance ecological validity as everyday life tasks typically are uninstructed and unpredictable. They then familiarized themselves with practice trials (eight practice trials in the single-task and 16 practice trials in the dual-task), followed by the experimental trials (16 trials in the single-task and 32 trials in the dual-task) per mapping. At the end of each task block, participants’ motivational state was assessed using DSSQ.

**Table 1:** Final participant allocation and study procedure by order condition

Setting Order	Initial Recruited (*N*)	Total Exclusion Due to Technical Issues	Total Exclusion Due to High Error Rates	Final Analysed (*N*)	Mapping Order (Initial Task)	Final Subgroup (*N*) Analyzed
Fixed Setting Order (Lab → DS)	39	14	4	21	Compatible → Incompatible	10
					Incompatible → Compatible	11
Post-hoc Counterbalanced (DS → Lab)	13	0	3	10	Compatible → Incompatible	6
					Incompatible → Compatible	4
Total Study Sample	52	14	7	31		31

Note. The table details the participant flow and final sample sizes after exclusions. It explicitly shows the counterbalancing procedure used for the modality mapping within each of the two setting order groups. The final analyzed sample size for the study is *N* = 31. Abbreviations: DS: Driving Simulator; Lab: Classical Laboratory

In the driving simulator setting, participants listened to the cover story (i.e., it was read to them by an experimenter) to embed the stimuli presented in the driving simulator in a more realistic scenario. After that, participants repeated the task procedure from the classical laboratory setting in the driving simulator.

### Data preprocessing

The dependent variables were reaction time and accuracy (only for correct trials), assessed from the vocal and manual responses in both single and dual tasks. Outlier exclusion was performed at the trial level, where reaction time values that were z-standardized beyond ± 3 standard deviations for each task, per mapping, and per setting were excluded (Aguinis et al., 2013).

In our preregistration, we specified that participants would be included if they completed at least 10 valid trials (out of 32 in the single-task and 64 in the dual-task condition) per condition. During data cleaning, we refined this threshold to five correct trials per task, per mapping, and per setting. We chose this more conservative criterion to maximize the usable sample while still ensuring sufficient data quality and reliability of estimates. Applying this criterion led to the exclusion of four participants from the initial sample (final *n* = 21). The same procedure was applied to the additional counterbalanced sample, resulting in the exclusion of three further participants (final *n* = 10). Taken together, a total of seven participants were excluded from subsequent analyses.

#### Manual responses

The reaction time of the manual responses was determined as the time interval from the stimulus onset to the first key press. Accuracy was determined as the percentage of correct responses relative to the total number of presented stimuli.

#### Vocal responses

 Firstly, a self-developed developed R script (Core Team, 2013) was used to preprocess the vocal responses given in the driving simulator. The continuous wave file was segmented, with each segment starting at the onset of a stimulus presentation and ending at the onset of the next stimulus. Secondly, the reaction time for each vocal responses from the driving simulator and classical laboratory settings were determined using a custom Matlab (MATLAB, 2019) script based on Reisner and Hinrichs (2016). The script utilized amplitude peak detection to measure speech onset. Specifically, reaction time was defined as the moment just before the highest peak where the amplitude fell below the average within a 6000 ms time window. Accuracy was calculated as the proportion of correct responses out of the total number of stimuli presented.

Dual-task cost in percentage was calculated as the relative difference between dual-task and single-task performance (i.e., dual-task cost = $\:\frac{single\:task\:-\:dual\:task}{single\:task}$ * 100) averaged across response modalities per modality mapping and setting. Reaction time cost was multiplied by −1 to have the same orientation as the accuracy cost. Positive values indicate dual-task costs compared to single-task performance for both accuracy and reaction time.

#### Statistical analysis

The data were analyzed statistically using RStudio version 4.3.1 (Core Team, 2013). The “lme4” package was employed for fitting linear mixed-effect models (LMM) (Bates et al., 2014). LMMs were applied to analyze the effect of the fixed effects of Setting (laboratory, driving simulator), Modality Mapping (compatible, incompatible) and their interaction on dual-task costs, separately for reaction time and accuracy. Age and Sex were defined as covariates and added as fixed effects to control for their potential influences on the dependent variables. Additionally, random intercepts for participants were introduced to account for the inherent variability across individuals.

Further, we conducted additional analysis to control for the effect of Setting Order (drive simulator first, classical laboratory first) and their interactions with Mapping and Setting on dual-task costs, separately for reaction time and accuracy. This served as a robustness check for our design. Age and Sex were included as covariates, and participant ID was treated as a random intercept.

All models were fitted using maximum likelihood (ML) estimation. Confidence intervals (CI), as well as generalized eta-squared (ηG²) were provided to interpret effects. We interpreted ηG² as follows: <0.06 = small, 0.06–0.14 = medium and > 0.14 = large effect size (Sánchez & Cervantes, 2016). Normal distribution of residuals was assessed using Kolmogorov-Smirnoff test. All residuals were normally distributed. Variance inflation factors (VIF) were examined for fixed effects and covariates to detect multicollinearity between the predictors and the dependent variables. Covariates with VIF > 5 were excluded from the model (Marcoulides & Raykov, 2019). A *p* <.05 was interpreted as statistically significant.

Finally, although task prioritization was not specified, a descriptive analysis of participants response order was performed to determine whether there was a consistent tendency to prioritize one task over another.

## Results

### Main findings

We excluded Setting-Order from the main analysis due to its lack of significant main and interaction effects (see below Control Analysis on Setting-Order Effects**)** to enhance model parsimony. Consequently, the analysis was conducted with data from 31 participants (mean age = 22.86 ± 2.04 years; women = 21, men = 10). The results are presented separately for reaction time and accuracy, each expressed as dual-task costs (cf. Supplementary data; Appendix F for raw reaction time and accuracy score. The raw reaction time and accuracy show the same pattern of results). Table 1 displays the means and standard deviations for reaction time and accuracy across single-task and dual-task conditions as well as dual-task costs, stratified by modality mapping and environmental settings.

### Reaction time

**Fig. 3:**
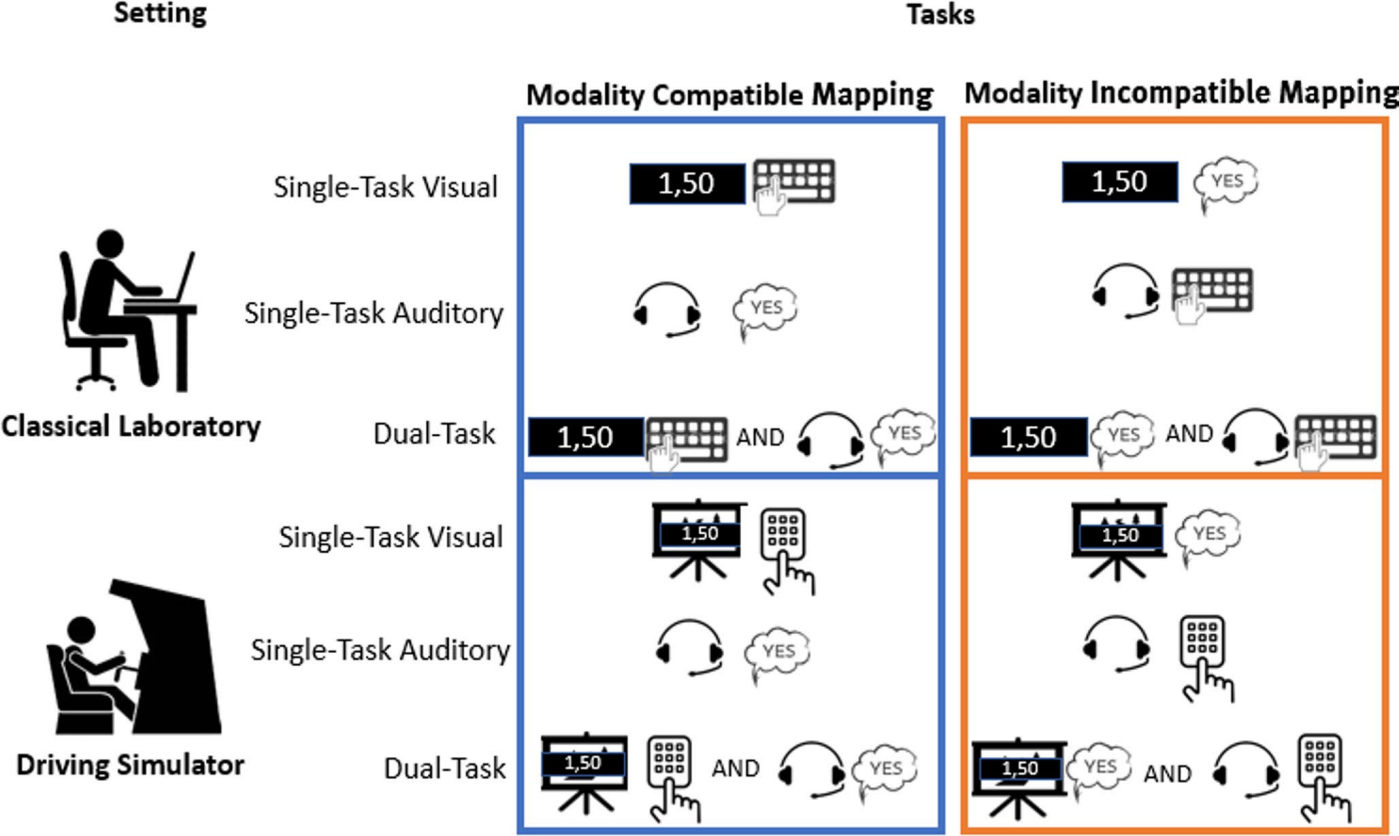
Experimental setting. Note. Overview of the experimental design: Conditions included modality compatible mappings and modality incompatible mappings. The experiment was conducted in two different settings: a classical laboratory and a driving simulator. In the initial design, the order of task performance was fixed, with participants always starting in the classical laboratory. To control for order effects, the design was later counterbalanced (post-hoc), such that some participants (*n* = 13) began in the driving simulator followed by the classical laboratory. Participants responded to the stimuli either vocally or manually. Stimuli comprised integers presented auditorily and decimal numbers presented visually. In the driving simulator, a cover story was introduced to give contextual meaning to the stimuli: integers were presented as traffic length while decimal numbers were presented as fuel (gas) prices to enhance the ecological validity of the scenario

The LMM results (cf. Table 2) revealed no main effect of Setting (*t* (90) *=* 0.74, *p =*.46, CI: [−7.68–17.16], ηG² = 0.31) indicating no difference in dual-task costs between the Classical Laboratory and Driving Simulator settings. In contrast, there was a significant main effect of Modality Mapping (*t* (90) *=* 4.17, *p* <.001, CI: [14.14–38.99], ηG² = 0.58), with higher dual-task costs associated with the modality incompatible mapping (compare Table 1; Fig. 3). Most importantly, there was a significant interaction between Setting and Modality Mapping (*t* (90) *=* 5.26, *p <.*001, CI: [29.89–65.04.94], ηG² = 0.24). Tukey’s HSD post-hoc test revealed that the difference between modality compatible mapping and modality incompatible mapping in dual-task costs was more pronounced in the Classical Laboratory setting (*ß* = 73.90, *SE* = 6.38, *p* <.001) compared to the Driving Simulator setting (*ß* = 26.60, *SE* = 6.38, *p* <.001). These results suggest that the modality compatibility effect was stronger in the classical laboratory setting than in the driving simulator setting.

**Table 2:** Descriptive statistics for reaction time (in ms) and accuracy (in %) for single task, dual-task and dual-task costs (in %) per setting

		Reaction Time		Accuracy	
	Setting	Modality Mapping		Modality Mapping	
		Compatible	Incompatible	Compatible	Incompatible
		***M*** **±** ***SD***	***M*** **±** ***SD***	***M*** **±** ***SD***	***M*** **±** ***SD***
ST	DS	898.16 ± 305.75	863.63 ± 239.36	96.07 ± 7.21	95.16 ± 8.94
	Lab	732.72 ± 253.23	686.07 ± 191.96	98.59 ± 2.26	98.39 ± 3.11
DT	DS	1201.07 ± 352.73	1384.73 ± 511.73	89.52 ± 17.69	92.54 ± 10.19
	Lab	1008.07 ± 322.37	1442.49 ± 646.48	95.67 ± 9.19	88.00 ± 10.73
DT cost	DS	33.85 ± 21.02	60.42 ± 29.48	6.56 ± 19.39	2.15 ± 12.85
	Lab	38.60 ± 24.03	112.64 ± 48.93	2.92 ± 9.48	10.52 ± 10.78

Note. Means (M) and standard deviation (SD) are presented. *ST* Single-task, *DT* Dual-task, *DS * Driving Simulator, *Lab* Classical Laboratory

### Accuracy

The LMM results (cf. Table 2) revealed no significant difference of Setting (*t* (90) *=* −1.09, *p =*.28, CI: [−10.16–2.88], ηG² = 0.01). This result indicates no difference in dual-task costs between the Classical Laboratory and Driving Simulator settings. Similarly, there was no significant difference between the Modality Mappings (*t* (90) *=* −1.32, *p =*.19, CI: [−10.93–2.11], ηG² < 0.01). However, a significant interaction between Setting and Modality Mapping was observed (*t* (90) *=* 2.54, *p =*.01, CI: [2.78–21.24], ηG² = 0.07). Turkey’s HSD post-hoc test revealed that the difference in dual-task costs between the modality compatible mapping and the modality incompatible mapping was larger in the Classical Laboratory setting (*ß* = 7.60, *SE* = 3.35, *p* =.03) compared to the Driving Simulator setting (*ß* = −4.41, *SE* = 3.35, *p* =.19). Similarly to the effects for reaction times, this finding suggests that the modality compatibility effect is higher in Classical Laboratory setting than Driving Simulator setting.

### Sensitivity analysis on setting-order effects

The sensitivity analysis was conducted post-hoc and was therefore intended as a manipulation check. The analysis revealed no significant main and interaction effects including Setting-Order (all *p* >.34). Similarly, for accuracy, the findings revealed no significant main or interaction effects including Setting Order (all *p* >.26) (cf. Supplementary data; Appendix D). These non-significant results suggest that the sequence in which the experimental settings were administered did not meaningfully influence dual-task performance nor the modality-compatibility effect.

**Fig. 4:**
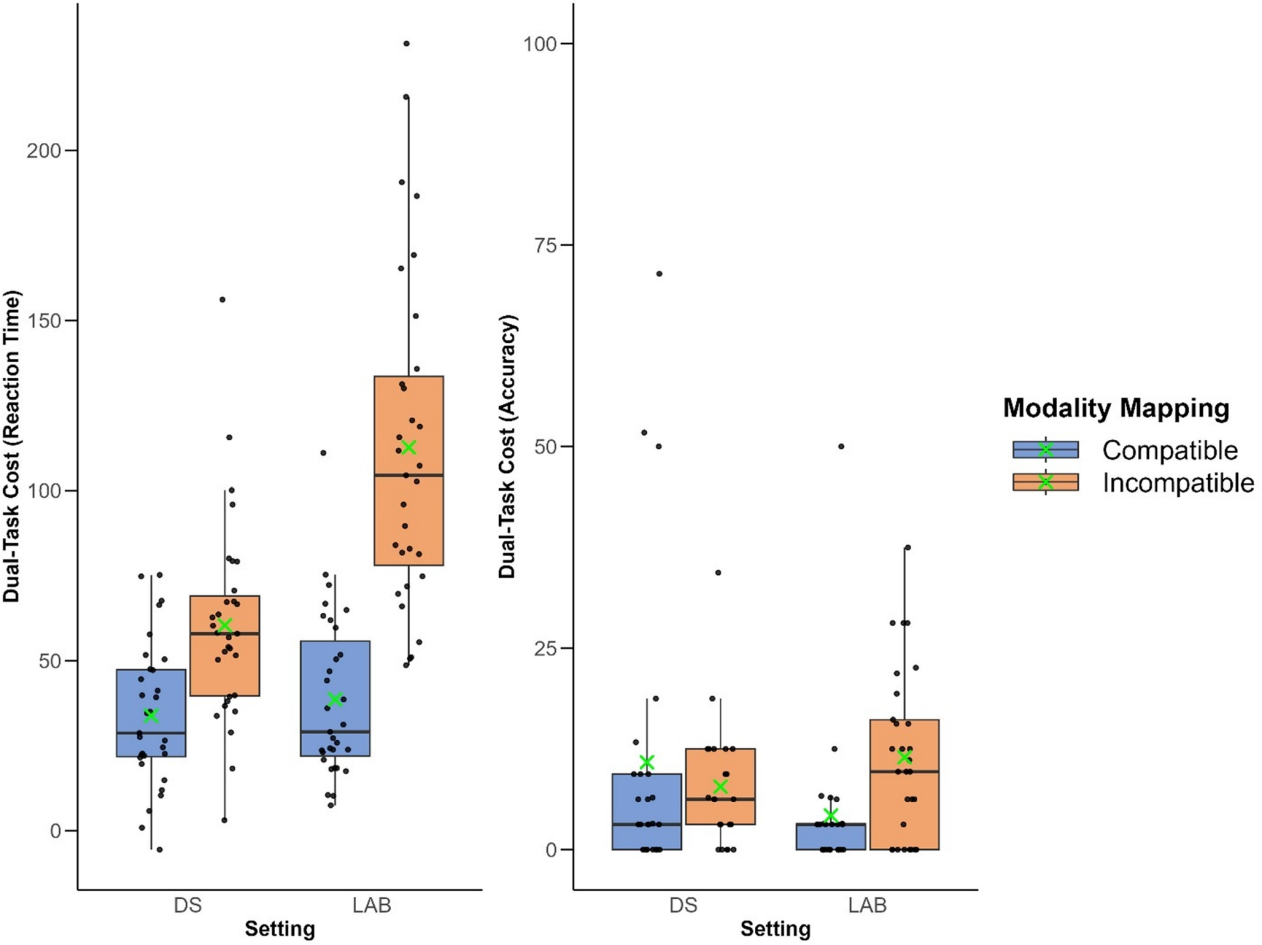
Dual-task costs across two distinct settings. Note. Boxplot showing the distribution of dual-task costs for reaction times and accuracy (both in percentage). Dots correspond to individuals per setting and modality mapping. The green ‘X’ indicates mean dual-task costs. DS = Driving Simulator; Lab = Classical Laboratory. Positive values indicate dual-task costs, i.e., lower performance on dual-task costs compared to single task, whereas a value of zero indicates the same performance level for single and dual tasks

Finally, descriptive analysis of participants’ response order revealed that in the Driving Simulator setting, under modality compatible mappings, participants responded first to the visual task in 84.6% of dual-task trials (*n* = 662), whereas the auditory task was prioritized in 15.4% of the dual-task trials (*n* = 121). A similar trend emerged in the classical laboratory setting, where the visual task was prioritized in 84.5% of dual-task trials (*n* = 667), and the auditory task in 15.5% (*n* = 122). In contrast, under modality incompatible mappings in the driving simulator, participants prioritized the auditory task in 69.1% of trials (*n* = 518), while the visual task was prioritized in 30.9% (*n* = 232). A comparable pattern was observed in the Classical Laboratory setting, with auditory prioritization at 56.2% (*n* = 402) and visual prioritization at 43.8% (*n* = 313). Interestingly, these findings suggest that task prioritization strategies were dependent on the response modality —participants preferred to respond manually first (which is the visual task in the modality compatible condition and the auditory task in the modality incompatible condition) and then vocally, independent of modality mapping and type of scenario (cf. supplementary data; Appendix H for inferential statistics) (Fig. 4 and Table 3).

**Table 3:** LMM results for reaction time and accuracy

Predictors	Coefficient	Std. Error	t value	*P*	95% CI Lower	95% CI Upper	ηG²
Reaction time							
Intercept	−30.27	48.86	−0.62	0.54	−124.15	63.62	
Setting (Lab)	4.74	6.38	0.74	0.46	−7.68	17.16	0.31
Mapping (Incompatible)	26.57	6.38	4.17	< 0.001***	14.14	38.99	0.58
Age	2.65	2.12	1.25	0.22	−1.43	6.73	0.05
Sex(M)	12.97	9.81	1.32	0.20	−5.88	31.81	0.06
Setting*Mapping	47.47	9.02	5.26	< 0.001***	29.89	65.04	0.24
Accuracy							
Intercept	7.93	15.49	0.51	0.61	−21.84	37.69	
Setting (Lab)	−3.64	3.35	−1.09	0.28	−10.16	2.88	0.01
Mapping (Incompatible)	−4.41	3.35	−1.32	0.19	−10.93	2.11	< 0.01
Age	−0.01	0.67	−0.02	0.99	−1.30	1.27	< 0.01
Sex(M)	−4.26	3.09	−1.38	0.18	−10.20	1.69	0.06
Setting*Mapping	12.01	4.74	2.54	0.01*	2.78	21.24	0.07

Note. Setting includes two levels (driving simulator, laboratory). Mapping includes two levels (compatible, incompatible). Age and Sex were included as covariates. P-values represent statistical significance. ***: p-value < 0.001, **: p-value < 0.01, *: p-value <. 05. CI = Confidence Interval; ηG² = generalized eta-squared. (cf. for analysis on raw reaction time and accuracy score see Supplementary data; Appendix F)

## Discussion

The present study investigated the effect of modality compatibility on the magnitude of dual-task costs between two different experimental settings: a classical laboratory and a driving simulator. Our findings revealed that dual-task costs for reaction time were significantly higher in modality incompatible mappings compared to modality compatible mappings, regardless of the setting. In contrast, no significant differences in dual-task costs for accuracy were observed between modality mappings, which is consistent with previous literature (Hazeltine et al., 2006). This outcome is likely attributable to the absence of a speed–accuracy trade-off, as participants appeared to maintain accuracy across conditions (cf. Supplementary Data; Appendix C). In view of this, our planned hierarchical drift-diffusion modeling (HDDM), which had been preregistered to account for potential speed–accuracy trade-offs, was not conducted and is therefore omitted. Furthermore, there was no significant difference in dual-task costs between settings for either outcome measure (reaction time, accuracy). However, a significant interaction between setting and modality mapping was found for both reaction time and accuracy, indicating that the effect of modality incompatibility mappings on dual-task costs was more pronounced in the classical laboratory compared to the driving simulator. Taken together, these results replicate previous findings on modality compatibility, showing that dual-task costs are higher under modality incompatible mappings than modality compatible mappings (Hazeltine et al., 2006; Mueckstein et al., 2022; Ruthruff et al., 2006; Stelzel & Schubert, 2011; Stelzel et al., 2006). Additionally, the results also confirm our hypothesis that the impact of modality compatibility is greater in the classical laboratory than in the driving simulator.

The finding of more pronounced dual-task costs under modality incompatible mappings compared to modality compatible mappings aligns with previous research on modality compatibility effects in dual-task paradigms (Hazeltine et al., 2006; Mueckstein et al., 2022; Stelzel & Schubert, 2011). This difference has been discussed as increased interference and processing demands due to overlapping sensory feedback in modality incompatible mappings (Navon & Miller, 1987). In contrast, modality compatible mappings facilitate more efficient processing because sensory input and response-related feedback align within each task, reducing crosstalk (Logan & Gordon, 2001; Schacherer & Hazeltine, 2021).

However, there was no significant difference in general dual-task costs between the classical laboratory and the driving simulator settings. This contradicts our initial hypothesis that multisensory integration and contextual cues in the driving simulator would facilitate more efficient dual-tasking, thus reducing dual-task cost. In contrast, this finding rather underscores the robustness of dual task interference across different experimental settings, suggesting that multitasking-related cognitive challenges extend from controlled laboratory conditions to real-world scenarios such as driving while performing secondary tasks (Asuako et al., 2025; Janouch et al., 2018; Wechsler et al., 2018). Nonetheless, previous studies have shown that multisensory integration and contextual cues enhance information processing, potentially mitigating dual-task costs in complex real-world tasks compared to simplified, isolated tasks in laboratory settings (Bailey et al., 2018; Cornelio et al., 2021; Ho et al., 2007). In contrast to these findings, our study involved young adults who were front seat passengers and therefore engaged with the simulator in a more passive role rather than performing as active drivers. It is conceivable that the benefits of multisensory integration would have been more pronounced if participants were required to actively drive, as this would more directly engage them in the integration of sensory information and driving-related decision-making. Thus, while our current data and design did not show overall setting differences, they offer valuable insight into how such cues may support passengers’ cognitive engagement, and future studies might further explore whether active driving further amplifies these effects. Although no overall setting effect was observed, a more nuanced pattern emerged when considering the interaction with modality compatibility.

Importantly, when considering the interaction between setting and modality compatibility, the difference in dual-task costs between modality incompatible mappings and modality compatible mappings was more pronounced in the classical laboratory setting than in the driving simulator. This pattern suggests that, although the simulator did not reduce overall dual-task costs, multisensory integration and contextual cues may still offer compensatory support for modality-based crosstalk. Specifically, the simulator’s greater task complexity, dynamic environment, and multisensory integration cues (Schuch et al., 2020) likely supported more efficient processing. Previous research has shown that multisensory environments facilitate sensory integration by engaging modality-specific brain regions, which in turn help segregate tasks, reduce interference, and enhance performance even under complex conditions (Laurienti et al., 2006). This may explain the relatively improved performance in modality incompatible mappings: reduced crosstalk and more effective perception-action coupling (Jeong et al., 2024). For example, drivers often respond more efficiently to hazards at intersections by integrating visual information (e.g., road signs) with auditory cues (e.g., Navigation systems; Ho et al., 2007). Moreover, real-world contextual cues in the driving simulator, such as the need to avoid lengthy traffic or hefty fuel prices, may enhance predictive processing. This enables participants to better anticipate sensory consequences, allocate attention more efficiently, and coordinate responses more effectively (Hommel, 2009; Prinz, 1997).This may mitigate the disadvantages of modality incompatible mappings by leveraging real-world constraints to improve performance (Bailey et al., 2018; Cornelio et al., 2021). In contrast, the classical laboratory setting presents a more abstract representation of tasks and fewer contextual cues. This lack of real-world sensory integration may amplify modality incompatibility effects, as participants rely solely on task-internal representation rather than external multisensory feedback, consequently increasing crosstalk (Wirth et al., 2020). Taken together, these findings suggest that although dual-task interference persists across settings, contextual and multisensory support in the simulator may attenuate the disadvantages of modality incompatible mappings.

### Strengths and limitations

Our study is the first to investigate the modality compatibility effect in a more realistic everyday scenario, effectively enhancing the ecological validity of our experiment and transfer of our result to similar real-life scenarios. That is, our results suggest that the modality compatibility effect might be generalized from a classical laboratory task to a real-life task such as driving, albeit reduced in size compared to the classical lab setting.

A limitation of the present study concerns the use of a cover story only in the driving simulator condition. While the cover story was introduced to enhance psychological fidelity and participant engagement in the naturalistic environment (De Winter et al., 2007), its absence in the classical laboratory condition introduces a potential confound. Specifically, the observed differences between settings may not solely reflect environmental or task-based contrasts, but could also be partially driven by differences in contextual framing. Without a fully crossed 2 × 2 design that independently manipulates setting (driving simulator vs. classical laboratory) and framing (narrative vs. neutral), it is not possible to isolate the effects of setting from those of narrative context. This limits the causal interpretability of setting-related effects. Future studies should aim to systematically vary contextual framing across different experimental environments to better disentangle their individual and combined contributions to task performance.

## Conclusion

Overall, the results of the present study extend previous findings by providing evidence that the modality compatibility effect exists in realistic environments, such as driving—although the magnitude of the impact is reduced. This highlights the need to consider modality compatibility when designing assistive devices and certain ergonomic environments, such as driving settings. For instance, in an in-vehicle environment, navigation systems that provide spoken instructions and allow drivers to respond verbally through voice recognition systems create a modality compatible scenario. This prevents drivers from diverting their attention away from the road, thereby enhancing safe driving.

## Supplementary Information

Below is the link to the electronic supplementary material.

Supplementary Material 1 (DOCX 574 KB)

## Data Availability

The authors will make the raw data supporting the conclusions of this manuscript available upon reasonable request.

## References

[CR1] Aguinis, H., Gottfredson, R. K., & Joo, H. (2013). Best-practice recommendations for defining, identifying, and handling outliers. *Organizational Research Methods,**16*(2), 270–301.

[CR2] Asuako, P. A., Stojan, R., Bock, O., Mack, M., & Voelcker-Rehage, C. (2025). Multitasking: Does task-switching add to the effect of dual-tasking on everyday-like driving behavior? *Cognitive Research: Principles and Implications,**10*(1), 1–14. 39921816 10.1186/s41235-025-00611-y PMC11807033

[CR3] Bailey, H. D., Mullaney, A. B., Gibney, K. D., & Kwakye, L. D. (2018). Audiovisual integration varies with target and environment richness in immersive virtual reality. *Multisensory Research,**31*(7), 689–713. 31264608 10.1163/22134808-20181301

[CR4] Bates, D., Mächler, M., Bolker, B., & Walker, S. (2014). Fitting linear mixed-effects models using lme4. *arXiv preprint arXiv:1406.5823*.

[CR5] Bock, O., Drescher, U., Janouch, C., Haeger, M., van Winsum, W., & Voelcker-Rehage, C. (2019a). An experimental paradigm for the assessment of realistic human multitasking. *Virtual Reality*, *23*, 61–70.

[CR6] Bock, O., Drescher, U., Janouch, C., Haeger, M., van Winsum, W., & Voelcker-Rehage, C. (2019b). An experimental paradigm for the assessment of realistic human multitasking. *Virtual Reality,**23*(1), 61–70. 10.1007/s10055-018-0342-7

[CR7] Calhoun, V. D., & Pearlson, G. D. (2012). A selective review of simulated driving studies: Combining naturalistic and hybrid paradigms, analysis approaches, and future directions. *NeuroImage,**59*(1), 25–35. 21718791 10.1016/j.neuroimage.2011.06.037 PMC3197797

[CR8] Chaytor, N., & Schmitter-Edgecombe, M. (2003). The ecological validity of neuropsychological tests: A review of the literature on everyday cognitive skills. *Neuropsychology Review*, *13*, 181–197. 15000225 10.1023/b:nerv.0000009483.91468.fb

[CR9] Core Team, R., R (2013). *R: A Language and environment for statistical computing*. In: R foundation for statistical computing Vienna, Austria.

[CR10] Cornelio, P., Velasco, C., & Obrist, M. (2021). Multisensory integration as per technological advances: A review. *Frontiers in Neuroscience,**15*, Article 652611. 34239410 10.3389/fnins.2021.652611 PMC8257956

[CR11] De Winter, J. C., Wieringa, P., Dankelman, J., Mulder, M., Van Paassen, M., & De Groot, S. (2007). Driving simulator fidelity and training effectiveness. Proceedings of the 26th European annual conference on human decision making and manual control, Lyngby, Denmark.

[CR12] Deroy, O., Faivre, N., Lunghi, C., Spence, C., Aller, M., & Noppeney, U. (2016). The complex interplay between multisensory integration and perceptual awareness. *Multisensory Research*, *29*(6–7), 585–606. 27795942 10.1163/22134808-00002529 PMC5082728

[CR13] Faul, F., Erdfelder, E., Lang, A.-G., & Buchner, A. (2007). G* power 3: A flexible statistical power analysis program for the social, behavioral, and biomedical sciences. *Behavior Research Methods,**39*(2), 175–191. 17695343 10.3758/bf03193146

[CR14] Fintor, E., Stephan, D. N., & Koch, I. (2018). Emerging features of modality mappings in task switching: Modality compatibility requires variability at the level of both stimulus and response modality. *Psychological Research,**82*(1), 121–133. 10.1007/s00426-017-0875-5 28578525 10.1007/s00426-017-0875-5

[CR15] Greenwald, A. G. (1970). Sensory feedback mechanisms in performance control: With special reference to the ideo-motor mechanism. *Psychological Review,**77*(2), 73. 5454129 10.1037/h0028689

[CR16] Hazeltine, E., Ruthruff, E., & Remington, R. W. (2006). The role of input and output modality pairings in dual-task performance: Evidence for content-dependent central interference. *Cognitive Psychology*, *52*(4), 291–345. 16581054 10.1016/j.cogpsych.2005.11.001

[CR17] Ho, C., Reed, N., & Spence, C. (2007). Multisensory in-car warning signals for collision avoidance. *Human Factors*, *49*(6), 1107–1114. 18074709 10.1518/001872007X249965

[CR18] Hommel, B. (2009). Action control according to TEC (theory of event coding). *Psychological Research PRPF*, *73*, 512–526. 10.1007/s00426-009-0234-2 PMC2694931 19337749

[CR19] Hullman, J., & Diakopoulos, N. (2011). Visualization rhetoric: Framing effects in narrative visualization. *IEEE Transactions on Visualization and Computer Graphics*, *17*(12), 2231–2240. 22034342 10.1109/TVCG.2011.255

[CR20] Janouch, C., Drescher, U., Wechsler, K., Haeger, M., Bock, O., & Voelcker-Rehage, C. (2018). Cognitive-motor interference in an ecologically valid street crossing scenario. *Frontiers in Psychology,**9*, Article 602. 10.3389/fpsyg.2018.00602 29774001 10.3389/fpsyg.2018.00602 PMC5944128

[CR21] Jeong, S., Kim, J., & Lee, J. (2024). The differential effects of multisensory attentional cues on task performance in VR depending on the level of cognitive load and cognitive capacity. *IEEE Transactions on Visualization and Computer Graphics*. 10.1109/TVCG.2024.3372126 38437135 10.1109/TVCG.2024.3372126

[CR22] Kahneman, D. (1973). *Attention and effort* (Vol. 1063). Citeseer.

[CR23] Laurienti, P. J., Burdette, J. H., Maldjian, J. A., & Wallace, M. T. (2006). Enhanced multisensory integration in older adults. *Neurobiology of Aging,**27*(8), 1155–1163. 16039016 10.1016/j.neurobiolaging.2005.05.024

[CR24] Levy, J., & Pashler, H. (2001). Is dual-task slowing instruction dependent? *Journal of Experimental Psychology: Human Perception and Performance*, *27*(4), 862. 11518149

[CR25] Lien, M.-C., & Proctor, R. W. (2002). Stimulus-response compatibility and psychological refractory period effects: Implications for response selection. *Psychonomic Bulletin & Review,**9*(2), 212–238. 12120784 10.3758/bf03196277

[CR26] Logan, G. D., & Gordon, R. D. (2001). Executive control of visual attention in dual-task situations. *Psychological Review,**108*(2), 393–434. 10.1037/0033-295x.108.2.393 11381835 10.1037/0033-295x.108.2.393

[CR27] Marcoulides, K. M., & Raykov, T. (2019). Evaluation of variance inflation factors in regression models using latent variable modeling methods. *Educational and Psychological Measurement,**79*(5), 874–882. 31488917 10.1177/0013164418817803 PMC6713981

[CR28] MATLAB, H. (2019). version 9.6. 0 (R2019a). *Natick, Massachusetts*.

[CR29] Matthews, G., Szalma, J., Panganiban, A. R., Neubauer, C., & Warm, J. S. (2013). Profiling task stress with the dundee stress state questionnaire. *Psychology of stress: New research,**1*, 49–90.

[CR30] Miller, J. (2006). Backward crosstalk effects in psychological refractory period paradigms: Effects of second-task response types on first-task response latencies. *Psychological Research = Psychologische Forschung,**70*(6), 484–493. 16237555 10.1007/s00426-005-0011-9

[CR31] Mueckstein, M., Heinzel, S., Granacher, U., Brahms, M., Rapp, M. A., & Stelzel, C. (2022). Modality-specific effects of mental fatigue in multitasking. *Acta Psychologica,**230*, Article 103766. 10.1016/j.actpsy.2022.103766 36242924 10.1016/j.actpsy.2022.103766

[CR32] Navon, D., & Miller, J. (1987). Role of outcome conflict in dual-task interference. *Journal of Experimental Psychology. Human Perception and Performance,**13*(3), 435. 2958592 10.1037//0096-1523.13.3.435

[CR33] Navon, D., & Miller, J. (2002). Queuing or sharing? A critical evaluation of the single-bottleneck notion. *Cognitive Psychology,**44*(3), 193–251. 10.1006/cogp.2001.0767 11971632 10.1006/cogp.2001.0767

[CR34] Pashler, H. (1994). Dual-task interference in simple tasks: Data and theory. *Psychological Bulletin*, *116*, 220–244. 10.1037/0033-2909.116.2.220 7972591 10.1037/0033-2909.116.2.220

[CR35] Pashler, H., Harris, C. R., & Nuechterlein, K. H. (2008). Does the central bottleneck encompass voluntary selection of hedonically based choices? *Experimental Psychology*, *55*(5), 313–321. 10.1027/1618-3169.55.5.313 25116298 10.1027/1618-3169.55.5.313

[CR36] Prinz, W. (1997). Perception and action planning. *European Journal of Cognitive Psychology*, *9*(2), 129–154.

[CR37] Reisner, V., & Hinrichs, D. (2016). The Response Onset Tool (v1. 0.0)[Software]. *Zenodo. doi*, *10*.

[CR38] Roider, F., Rümelin, S., Pfleging, B., & Gross, T. (2017). The effects of situational demands on gaze, speech and gesture input in the vehicle. Proceedings of the 9th International Conference on Automotive User Interfaces and Interactive Vehicular Applications.

[CR39] Ruthruff, E., Hazeltine, E., & Remington, R. W. (2006). What causes residual dual-task interference after practice? *Psychological Research = Psychologische Forschung,**70*, 494–503. 16184395 10.1007/s00426-005-0012-8

[CR40] Sánchez, M. E. T., & Cervantes, R. J. M. (2016). Generalized eta squared for multiple comparisons on between-groups designs. *Psicothema,**28*(3), 340–345. 27448270 10.7334/psicothema2015.124

[CR41] Schacherer, J., & Hazeltine, E. (2020). Cue the effects: Stimulus-action effect modality compatibility and dual-task costs. *Journal of Experimental Psychology. Human Perception and Performance,**46*(4), 350. 32223289 10.1037/xhp0000719 PMC7472667

[CR42] Schacherer, J., & Hazeltine, E. (2021). Crosstalk, not resource competition, as a source of dual-task costs: Evidence from manipulating stimulus-action effect conceptual compatibility. *Psychonomic Bulletin & Review*, *28*(4), 1224–1232. 33689145 10.3758/s13423-021-01903-2

[CR43] Schaeffner, S., Koch, I., & Philipp, A. M. (2018). Sensory-motor modality compatibility in multitasking: The influence of processing codes. *Acta Psychologica,**191*, 210–218. 10.1016/j.actpsy.2018.09.012 30312892 10.1016/j.actpsy.2018.09.012

[CR44] Schuch, S., Bock, O., Freitag, K., & Moretti, L. (2020). Cognitive multitasking: Inhibition in task switching depends on stimulus complexity. *Journal of Cognition,**3*(1), Article 30. 10.5334/joc.115 33043240 10.5334/joc.115 PMC7528661

[CR45] Stelzel, C., & Schubert, T. (2011). Interference effects of stimulus–response modality pairings in dual tasks and their robustness. *Psychological Research = Psychologische Forschung,**75*(6), 476–490. 21811837 10.1007/s00426-011-0368-x

[CR46] Stelzel, C., Schumacher, E. H., Schubert, T., & D ‘Esposito, M. (2006). The neural effect of stimulus-response modality compatibility on dual-task performance: An fMRI study. *Psychological Research,**70*, 514–525. 16175414 10.1007/s00426-005-0013-7

[CR47] Stephan, D. N., & Koch, I. (2010). Central cross-talk in task switching: Evidence from manipulating input–output modality compatibility. *Journal of Experimental Psychology. Learning, Memory, and Cognition,**36*(4), 1075. 20565224 10.1037/a0019695

[CR48] Stephan, D. N., & Koch, I. (2016). Modality-specific effects on crosstalk in task switching: Evidence from modality compatibility using bimodal stimulation. *Psychological Research,**80*, 935–943. 26377338 10.1007/s00426-015-0700-y

[CR49] Stojan, R., Kaushal, N., Bock, O. L., Hudl, N., & Voelcker-Rehage, C. (2021). Benefits of higher cardiovascular and motor coordinative fitness on driving behavior are mediated by cognitive functioning: a path analysis [Original Research]. *Frontiers in Aging Neuroscience*, 13. 10.3389/fnagi.2021.686499. https://www.frontiersin.org/articles/10.3389/fnagi.2021.686499 PMC8277437 34267646

[CR50] Strayer, D. L., Turrill, J., Cooper, J. M., Coleman, J. R., Medeiros-Ward, N., & Biondi, F. (2015). Assessing cognitive distraction in the automobile. *Human Factors,**57*(8), 1300–1324. 10.1177/0018720815575149 26534847 10.1177/0018720815575149

[CR51] Wechsler, K., Drescher, U., Janouch, C., Haeger, M., Voelcker-Rehage, C., & Bock, O. (2018). Multitasking during simulated car driving: A comparison of young and older persons. *Frontiers in Psychology,**9*, Article 910. 10.3389/fpsyg.2018.00910 29962983 10.3389/fpsyg.2018.00910 PMC6013591

[CR52] Wirth, R., Koch, I., & Kunde, W. (2020). Localizing modality compatibility effects: Evidence from dual-task interference. *Journal of Experimental Psychology: Human Perception and Performance*, *46*(12), 1527. 32969688 10.1037/xhp0000873

[CR53] World-Medical-Association. (2013). World Medical Association Declaration of Helsinki: Ethical principles for medical research involving human subjects. *JAMA,**310*(20), 2191–2194. 24141714 10.1001/jama.2013.281053

