# Supplementary material for: Effect of modality compatibility on dual-task performance in a more naturalistic environment
Source: Psychol Res. 2026 Jan 27;90(1):21. doi: 10.1007/s00426-026-02238-0 (PMC12847215; doi:10.1007/s00426-026-02238-0)
Supplement: Supplementary file 1 — Supplementary Material 1 (DOCX 574 KB) [file 426_2026_2238_MOESM1_ESM.docx]

**Appendix A**

**Relationship between modality compatibility effect in different environments.**

**Figure A1**

*Correlation between the modality compatibility effect in different environments for reaction time*


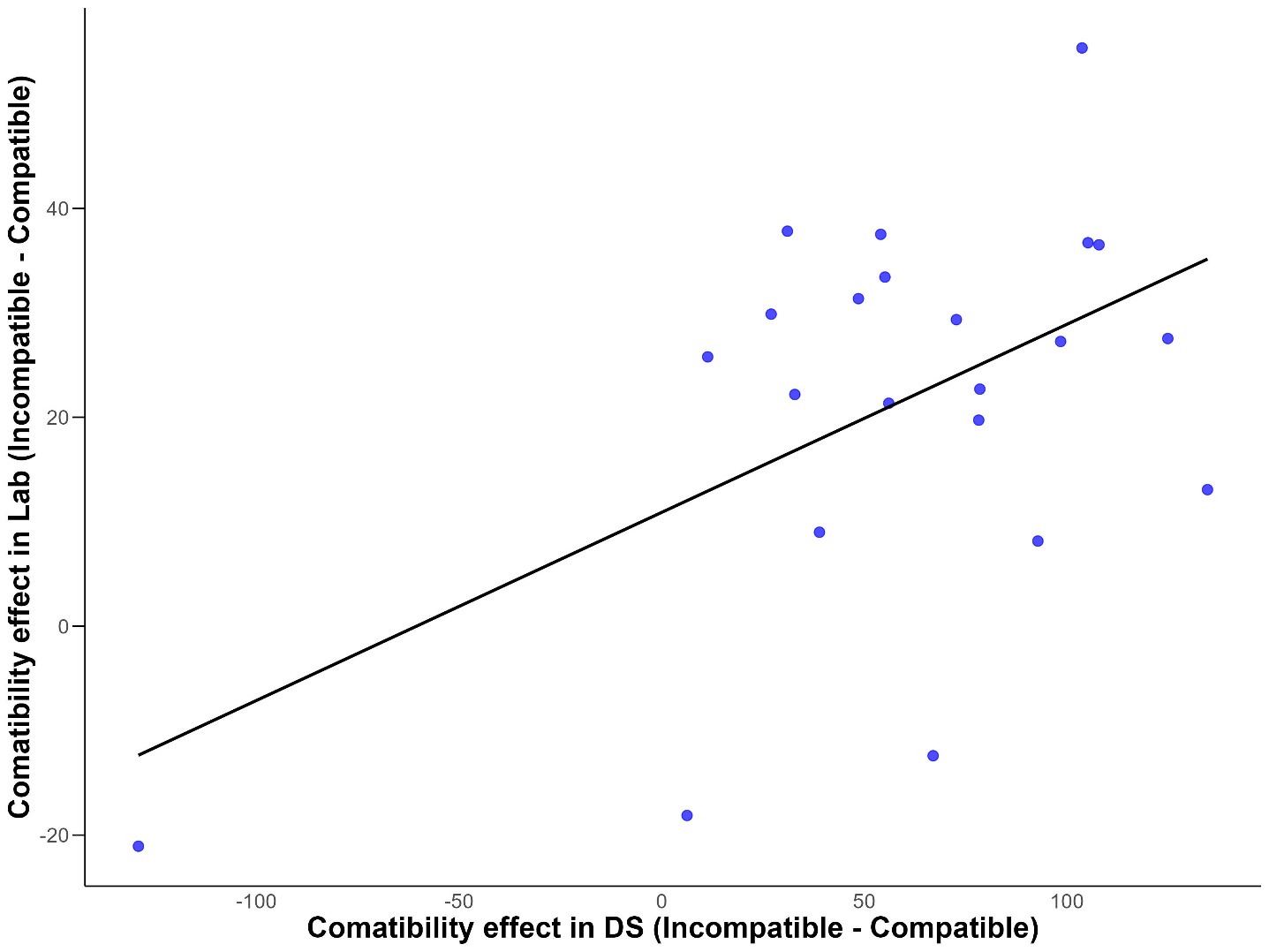


*Note.* Scatter plot showing the relationship between the modality compatibility effect in both environments. DS = Driving simulator; Lab = Laboratory

**Figure A2**

*Correlation between the modality compatibilty effect in different environments for response accuracy*


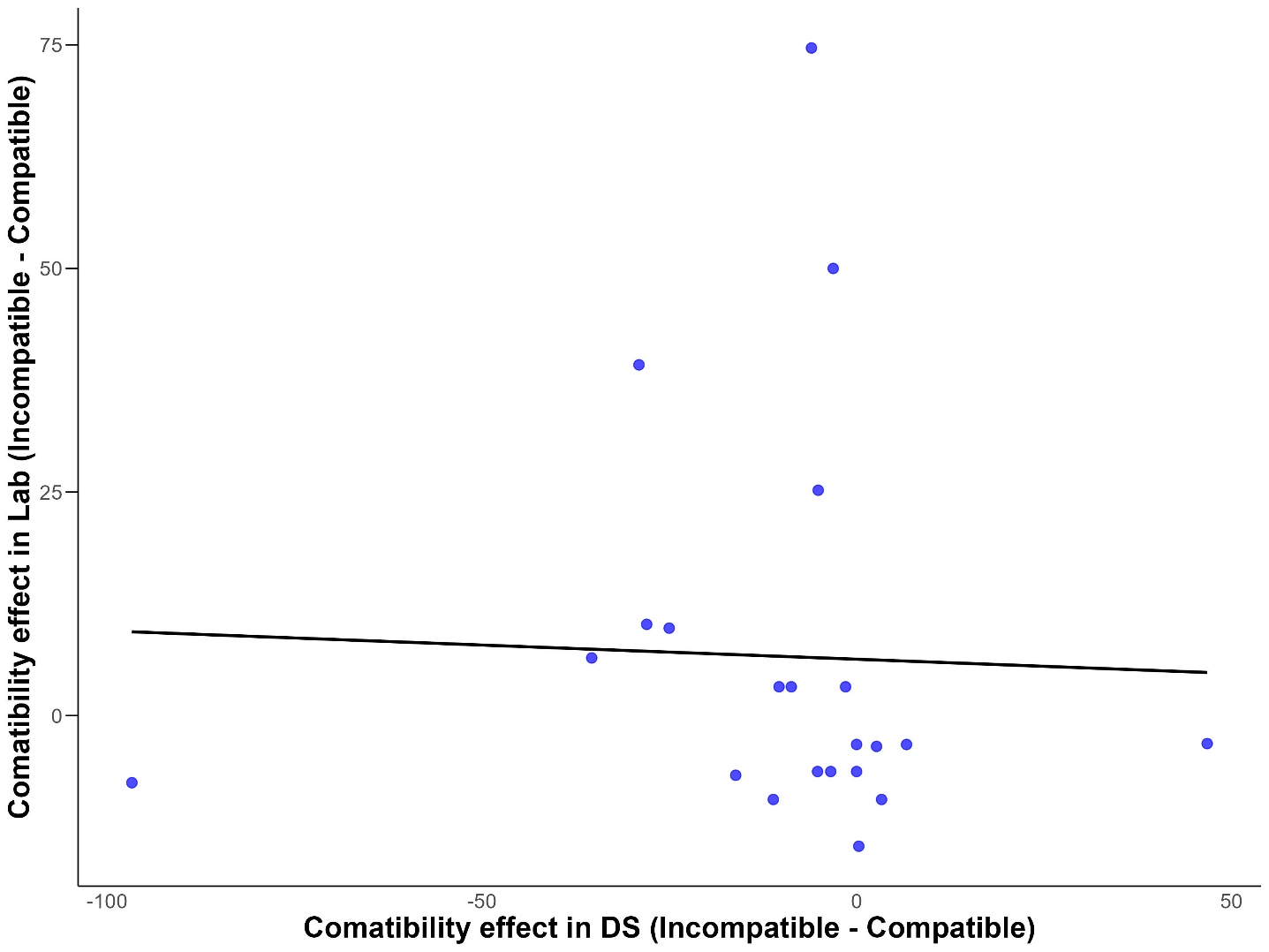


*Note.* Scatter plot showing the relationship between the modality compatibility effect in both environments. DS = Driving simulator; Lab = Laboratory

**Appendix B**

**Raw scores of reaction time and response for individual participants in both environments**

**Figure B1**

*Raw scores of reaction time*


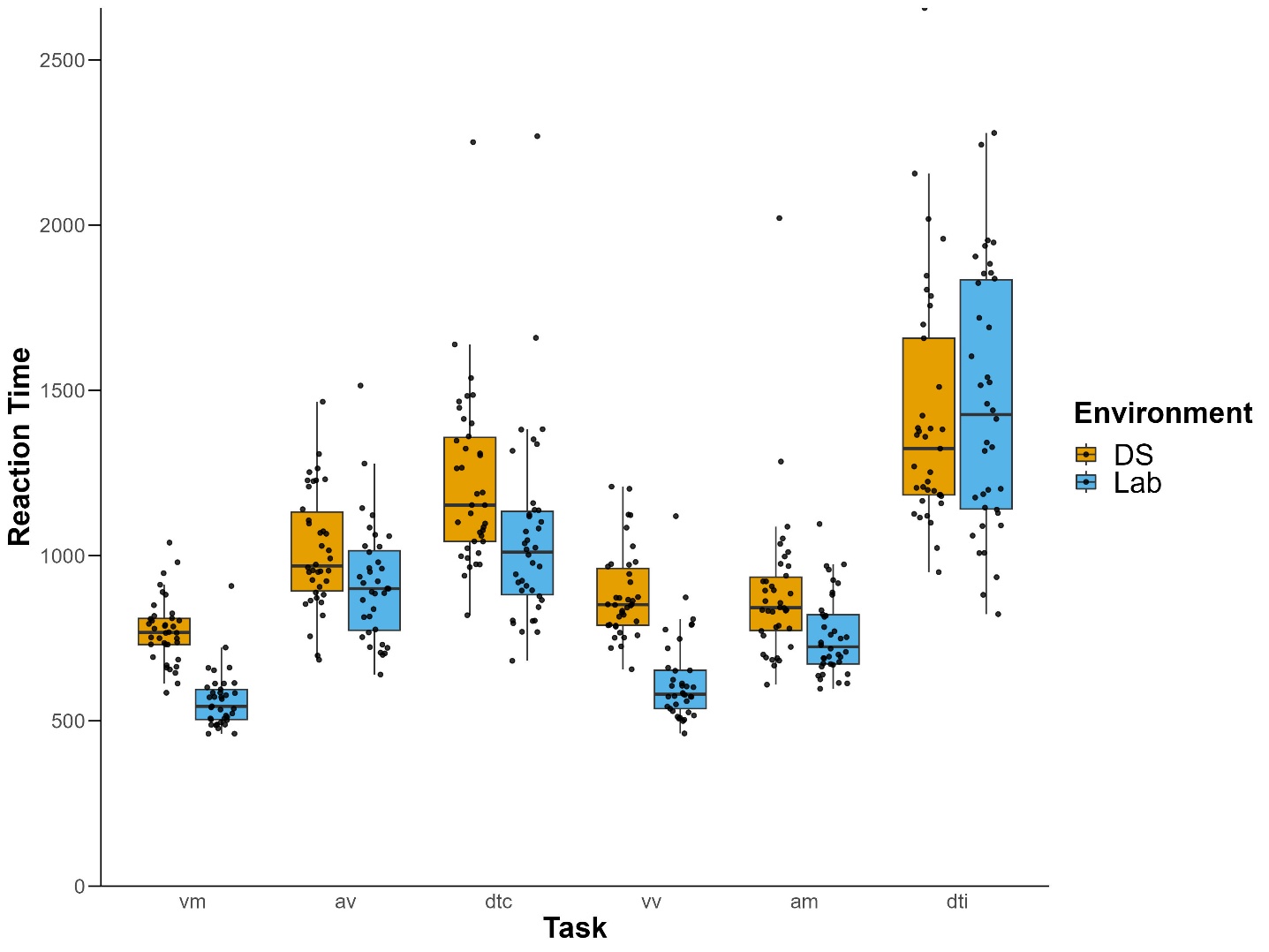


*Note. Boxplot showing the distribution of reaction times per task for individual participants. DS = Driving simulator; LAB = Laboratory; am = auditory-manual; av = auditory-vocal; dtc = dual-task compatible; dti = dual-task incompatible; vm = visual-manual; vv = visual-vocal.*

**Figure B2**

*Raw scores of response accuracy*


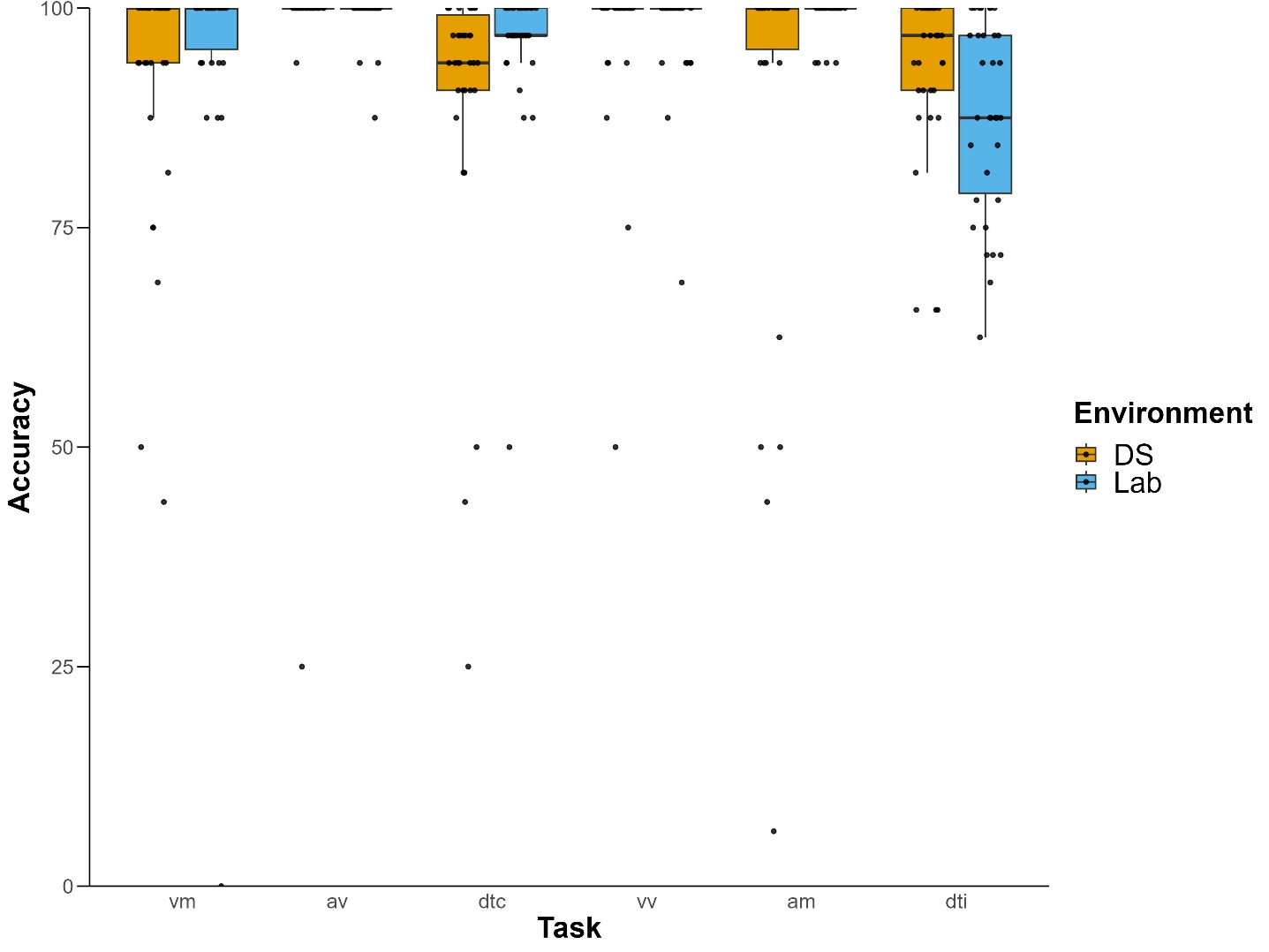


*Note. Boxplot showing the distribution of response accuracy per task for individual participants. DS = Driving simulator; LAB = Laboratory; am = auditory-manual; av = auditory-vocal; dtc = dual-task compatible; dti = dual-task incompatible; vm = visual-manual; vv = visual-vocal.*

**Appendix C**

**Relationship between Reaction Time and Accuracy (Speed-Accuracy Tradeoff)**

**
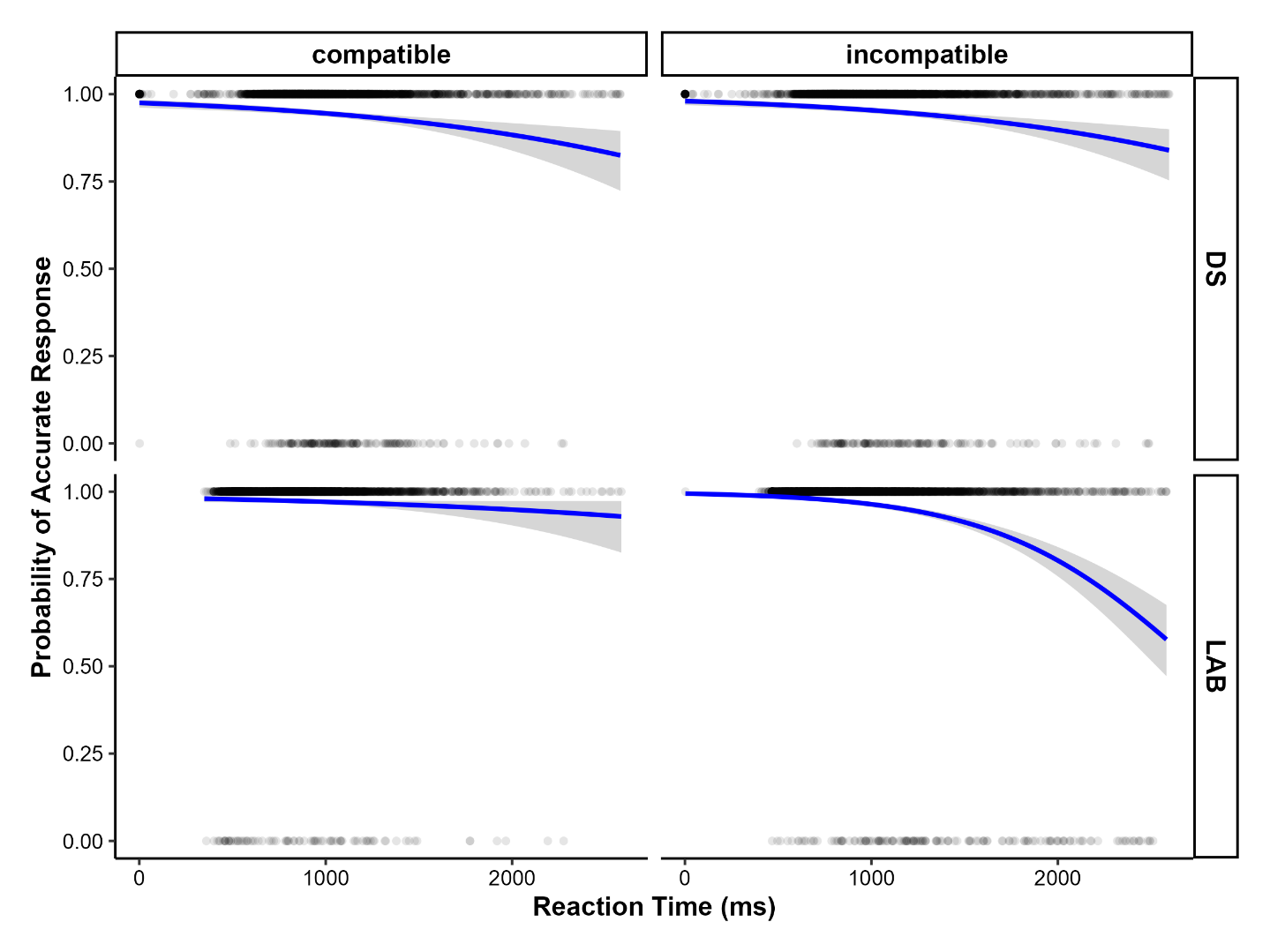
**

*Note*. The scatter plot shows a negative relationship between Reaction Time and Accuracy, indicating that as participants' response times decrease (i.e., they respond faster), their accuracy tends to increase. This pattern is counter to the typical speed-accuracy tradeoff, where faster responses are generally associated with a decrease in accuracy.

**Appendix D**

*LMM results for reaction time and accuracy with setting order as a factor****.***

| Predictors | Coefficient | Std. Error | t value | P | 95% CI Lower | 95% CI Upper | **ηG²** |
| --- | --- | --- | --- | --- | --- | --- | --- |
| Reaction time | | | | | | | |
| Intercept | -35.42 | 46.62 | -0.76 | .45 | -123.71 | 52.86 |  |
| Mapping (Incompatible) | 20.08 | 9.46 | 2.12 | .**04*** | 1.93 | 38.23 | 0.57 |
| Setting (Lab) | -0.43 | 9.46 | -0.05 | .96 | -18.58 | 17.72 | 0.28 |
| Order (Lab_First) | 0.74 | 11.39 | 0.07 | .95 | -20.76 | 22.25 | 0.09 |
| Age | 2.75 | 2.07 | 1.32 | 0.19 | -1.18 | 6.68 | 0.06 |
| Sex(M) | 20.77 | 9.41 | 2.21 | **0.04*** | 2.95 | 38.59 | 0.14 |
| Setting*Mapping | 35.82 | 13.37 | 2.68 | **.01**** | 10.14 | 61.49 | 0.22 |
| Mapping*Order | 11.04 | 12.03 | 0.92 | .36 | -12.06 | 34.13 | 0.05 |
| Setting*Order | 8.87 | 12.03 | 0.74 | .46 | -14.23 | 31.97 | 0.04 |
| Mapping*Setting*Order | 16.38 | 17.02 | 0.96 | .34 | -16.29 | 49.04 | <0.01 |
| Accuracy | | | | | | | |
| Intercept | 3.78 | 18.05 | 0.21 | .84 | -30.38 | 37.94 |  |
| Mapping (Incompatible) | -5.69 | 6.72 | -0.85 | .40 | -18.56 | 7.18 | 0.01 |
| Setting (Lab) | -6.76 | 6.72 | -1.01 | .32 | -19.63 | 6.11 | 0.01 |
| Order (Lab_First) | 7.26 | 6.19 | 1.18 | .24 | -4.49 | 19.00 | 0.14 |
| Age | -0.13 | 0.79 | -0.16 | .87 | -1.62 | 1.36 | <0.01 |
| Sex(M) | -3.02 | 3.58 | -0.85 | .41 | -9.79 | 3.75 | 0.02 |
| Setting*Mapping | 21.63 | 9.50 | 2.28 | **.03*** | 3.43 | 39.83 | 0.08 |
| Mapping*Order | 0.99 | 8.55 | 0.12 | .91 | -15.38 | 17.37 | <0.01 |
| Setting*Order | 3.28 | 8.55 | 0.38 | .70 | -13.09 | 19.66 | <0.01 |
| Mapping*Setting*Order | -8.74 | 12.09 | -0.72 | .47 | -31.90 | 14.41 | <0.01 |

*Note*. Setting includes two levels (driving simulator, laboratory). Mapping includes two levels (compatible, incompatible). Order includes two levels (DS_First, Lab_First) Age and Sex were included as covariates. P-values represent statistical significance. ***: p-value < .001, **: p-value < .01, *: p-value <. 05. CI = Confidence Interval; ηG² = general eta-squared. Abbreviation. DS: driving simulator; Lab: Classical laboratory

*Descriptive statistics for reaction time (in ms) and accuracy (in %) for single task, dual-task and dual-task costs per setting by order.*

|  |  |  | **Reaction Time** | | **Accuracy** | |
| --- | --- | --- | --- | --- | --- | --- |
|  | **Setting** | **Order** | **Modality Mapping** | | **Modality Mapping** | |
|  |  |  | **Compatible** | **Incompatible** | **Compatible** | **Incompatible** |
|  |  |  | ***M* ± *SD*** | ***M* ± *SD*** | ***M* ± *SD*** | ***M* ± *SD*** |
| ST | DS | DS_First | 927.31 ± 237.77 | 912.77 ± 202.83 | 0.94 ± 0.12 | 0.93 ± 0.14 |
|  |  | Lab_First | 890.47 ± 334.83 | 841.04 ± 253.86 | 0.96 ± 0.07 | 0.95 ± 0.10 |
|  | Lab | DS_First | 737.21 ± 242.56 | 696.47 ± 200.04 | 0.95 ± 0.14 | 0.99 ± 0.01 |
|  |  | Lab_First | 738.25 ± 259.72 | 690.13 ± 191.65 | 0.99 ± 0.02 | 0.98 ± 0.04 |
| DT | DS | DS_First | 1222.19 ± 382.48 | 1410.92 ± 548.39 | 0.93 ± 0.05 | 0.95 ± 0.06 |
|  |  | Lab_First | 1186.82 ± 323.13 | 1382.24 ± 491.59 | 0.88 ± 0.21 | 0.91 ± 0.12 |
|  | Lab | DS_First | 988.07 ± 354.24 | 1310.63 ± 588.56 | 0.98 ± 0.02 | 0.90 ± 0.09 |
|  |  | Lab_First | 1035.51 ± 309.29 | 1521.41 ± 660.13 | 0.95 ± 0.11 | 0.86 ± 0.12 |
| DT cost | DS | DS_First | 33.85 ± 27.42 | 53.93 ± 32.20 | -0.24 ± 5.10 | -2.85 ± 5.70 |
|  |  | Lab_First | 32.99 ± 18.00 | 64.10 ± 28.58 | 7.64 ± 8.90 | 3.30 ± 6.20 |
|  | Lab | DS_First | 33.42 ± 23.89 | 89.32 ± 41.27 | -3.57 ± 7.40 | 8.96 ± 4.80 |
|  |  | Lab_First | 41.43 ± 24.38 | 124.74 ± 52.24 | 4.07 ± 6.30 | 12.30 ± 7.90 |

*Note.* Means (M) and standard deviation (SD) are presented. ST = Single Task; DT = Dual Task; DS = Driving Simulator; Lab = Classical Laboratory

**Appendix E**

*LMM results for restricted sample (N = 16) (cf. Figure B2)*

| Predictors | Coefficient | Std. Error | t value | P | 95% CI Lower | 95% CI Upper | **ηG²** |
| --- | --- | --- | --- | --- | --- | --- | --- |
| Reaction time | | | | | | | |
| Intercept | 1.05 | 92.32 | 0.01 | .99 | -171-67 | 173.77 |  |
| Setting (Lab) | 5.98 | 11.36 | 0.53 | .60 | -16.00 | 27.96 | 0.35 |
| Mapping (Incompatible) | 32.03 | 11.36 | 2.82 | **0.01**** | 10.05 | 54.02 | 0.61 |
| Age | 1.28 | 4.07 | 0.31 | .76 | -6.34 | 8.90 | <0.01 |
| Sex(M) | 25.66 | 20.86 | 1.23 | .24 | -13.39 | 64.70 | 0.11 |
| Setting*Mapping | 64.99 | 16.07 | 4.04 | **<.001***** | 33.90 | 96.08 | 0.28 |
| Accuracy | | | | | | | |
| Intercept | -11.42 | 20.17 | -0.57 | .58 | -49.15 | 26.32 |  |
| Setting (Lab) | -0.27 | 3.54 | -0.08 | .94 | -7.13 | 6.58 | 0.09 |
| Mapping (Incompatible) | -1.06 | 3.54 | -0.30 | .77 | -7.92 | 5.79 | 0.12 |
| Age | 0.50 | 0.89 | 0.56 | .58 | -1.16 | 2.16 | 0.03 |
| Sex(M) | -4.15 | 4.55 | -0.91 | .38 | -12.66 | 4.35 | 0.07 |
| Setting*Mapping | -9.89 | 5.01 | -1.97 | .06 | -19.58 | -0.19 | 0.08 |

*Note.* Setting includes two levels (driving simulator, laboratory). Mapping includes two levels (compatible, incompatible). Age and Sex were included as covariates. P-values represent statistical significance. ***: p-value < .001, **: p-value < .01, *: p-value <. 05. CI = Confidence Interval; ηG² = general eta-squared.

**Appendix F**

*LMM results for raw reaction time and accuracy*

| Predictors | Coefficient | Std. Error | t value | P | 95% CI Lower | 95% CI Upper | **ηG²** |
| --- | --- | --- | --- | --- | --- | --- | --- |
| Reaction time | | | | | | | |
| Intercept | 581.09 | 463.88 | 1.25 | .23 | -444.32 | 1607.09 |  |
| Setting (Lab) | -152.42 | 26.64 | -5.72 | <.001*** | -204.97 | -99.85 | 0.40 |
| Mapping (Incompatible) | 171.93 | 31.26 | 5.50 | **<.001***** | 110.06 | 233.80 | 0.53 |
| Task Type (ST) | -338.39 | 16.67 | -20.31 | **<.001***** | -371.02 | -305.69 | 0.41 |
| Age | 28.61 | 20.23 | 1.41 | .17 | -16.18 | 73.38 | 0.10 |
| Sex(M) | -146.33 | 114.10 | -1.28 | .22 | -413.53 | 120.47 | 0.08 |
| Setting*Mapping | 260.43 | 23.82 | 10.93 | **<.001***** | 213.72 | 307.08 | 0.01 |
| Mapping*Task Type | -205.99 | 23.66 | -8.71 | **<.001***** | -252.39 | -159.65 | 0.07 |
| Setting*Task Type | 18.64 | 23.61 | 0.79 | **.**43 | -27.74 | 64.81 | <0.01 |
| Setting*Mapping*Task Type | -275.76 | 33.54 | -8.22 | **<.001***** | -341.38 | -209.91 | 0.01 |
| Accuracy | | | | | | | |
| Intercept | 0.93 | 0.15 | 6.37 | **<.001***** | 0.65 | 1.21 |  |
| Setting (Lab) | 0.06 | 0.02 | 2.57 | **.02*** | -0.01 | 0.11 | <0.01 |
| Mapping (Incompatible) | 0.04 | 0.02 | 1.62 | .11 | -0.01 | 0.08 | 0.05 |
| Task Type | 0.09 | 0.01 | 7.61 | **<.001***** | 0.07 | 0.12 | 0.03 |
| Age | -0.003 | 0.006 | -0.33 | .75 | -0.01 | 0.01 | <0.01 |
| Sex(M) | 0.02 | 0.04 | 0.50 | .62 | -0.05 | 0.09 | 0.01 |
| Setting*Mapping | -0.14 | 0.02 | -7.87 | **<.001***** | -0.17 | -0.10 | <0.01 |
| Mapping*Task Type | -0.06 | 0.02 | -3.18 | .08 | 0.09 | -0.02 | <0.01 |
| Setting*Task Type | -0.06 | 0.02 | -3.26 | .42 | 0.08 | -0.02 | <0.01 |
| Setting*Mapping*Task Type | 0.16 | 0.03 | 6.34 | **<.001***** | 0.11 | 0.21 | <0.01 |

*Note.* Setting includes two levels (driving simulator, laboratory). Mapping includes two levels (compatible, incompatible). Age and Sex were included as covariates. P-values represent statistical significance. ***: p-value < .001, **: p-value < .01, *: p-value <. 05. CI = Confidence Interval; ηG² = general eta-squared.

**Appendix G**

*LMM results for reaction time and accuracy with motivation as covariate***.**

| Predictors | Coefficient | Std. Error | t value | P | 95% CI Lower | 95% CI Upper | **ηG²** |
| --- | --- | --- | --- | --- | --- | --- | --- |
| Reaction time | | | | | | | |
| Intercept | 757.83 | 617.45 | 1.23 | .24 | -444.32 | 1607.09 |  |
| Setting (Lab) | -152.41 | 26.65 | -5.72 | **<.001***** | -204.97 | 99.85 | 0.40 |
| Mapping (Incompatible) | 172.01 | 31.25 | 5.50 | **<.001***** | 110.06 | 233.80 | 0.53 |
| Task Type (ST) | -338.37 | 16.67 | -20.31 | **<.001***** | -371.02 | -305.69 | 0.41 |
| Age | 32.98 | 20.66 | 1.60 | .13 | -16.18 | 73.38 | 0.10 |
| Sex(M) | -184.14 | 116.50 | -1.58 | .13 | -413.53 | 120.47 | 0.08 |
| Motivation | -43.06 | 60.10 | -0.72 | .48 | -201.23 | 114.52 | 0.03 |
| Setting*Mapping | 260.38 | 23.82 | 10.93 | **<.001***** | 213.72 | 307.08 | 0.01 |
| Mapping*Task Type | -206.07 | 23.66 | -8.71 | **<.001***** | -252.39 | -159.65 | 0.07 |
| Setting*Task Type | 18.56 | 23.61 | 0.79 | **.43** | -27.74 | 64.81 | <0.01 |
| Setting*Mapping*Task Type | -275.64 | 33.54 | -8.22 | **<.001***** | -341.38 | -209.91 | 0.01 |
| Accuracy | | | | | | | |
| Intercept | 0.96 | 0.19 | 4.94 | **<.001***** | 0.65 | 1.21 |  |
| Setting (Lab) | 0.06 | 0.02 | 2.57 | **02*** | 0.01 | 0.11 | <0.01 |
| Mapping (Incompatible) | 0.04 | 0.02 | 1.62 | .11 | -0.01 | 0.08 | 0.05 |
| Task Type | 0.09 | 0.01 | 7.61 | **<.001***** | 0.07 | 0.12 | 0.03 |
| Age | -0.003 | 0.006 | -0.33 | .75 | -0.01 | 0.01 | <0.01 |
| Sex(M) | 0.02 | 0.04 | 0.51 | .64 | -0.05 | 0.09 | 0.01 |
| Motivation | -0.01 | 0.02 | -0.26 | .81 | -0.04 | 0.03 | <0.01 |
| Setting*Mapping | -0.14 | 0.02 | -7.87 | **<.001***** | -0.17 | -0.10 | <0.01 |
| Mapping*Task Type | -0.06 | 0.02 | -3.18 | .08 | 0.09 | -0.02 | <0.01 |
| Setting*Task Type | -0.06 | 0.02 | -3.26 | .42 | 0.08 | -0.02 | <0.01 |
| Setting*Mapping*Task Type | 0.16 | 0.03 | 6.34 | **<.001***** | 0.11 | 0.21 | <0.01 |

*Note.* Setting includes two levels (driving simulator, laboratory). Mapping includes two levels (compatible, incompatible). Age and Sex were included as covariates. P-values represent statistical significance. ***: p-value < .001, **: p-value < .01, *: p-value <. 05. CI = Confidence Interval; ηG² = general eta-squared.

**Appendix H**

*Mixed-effect logistic regression analysis of response order*

| Predictors | Estimate | Std. Error | z value | P value | Odds Ratio |
| --- | --- | --- | --- | --- | --- |
| Response order |  |  |  |  |  |
| Intercept | -0.36 | 0.12 | -3.03 | **.01**** | 0.64 |
| Setting (Lab) | -0.04 | 0.08 | -0.46 | .65 | 0.96 |
| Mapping (Incompatible) | -0.44 | 0.08 | -5.24 | **<.001***** | 0.56 |
| Setting*Mapping | -0.21 | 0.12 | -1.76 | .07 | 0.79 |

*Note.* Setting includes two levels (driving simulator, laboratory). Mapping includes two levels (compatible, incompatible). Age and Sex were included as covariates. P-values represent statistical significance. ***: p-value < .001, **: p-value < .01, *: p-value <. 05
